# High-dose *Mycobacterium tuberculosis* aerosol challenge cannot overcome BCG-induced protection in Chinese origin cynomolgus macaques; implications of natural resistance for vaccine evaluation

**DOI:** 10.1038/s41598-021-90913-0

**Published:** 2021-06-10

**Authors:** Laura Sibley, Andrew D. White, Karen E. Gooch, Lisa M. Stevens, Rachel Tanner, Ashley Jacobs, Owen Daykin-Pont, Fergus Gleeson, Anthony McIntyre, Randall Basaraba, Simon Clark, Graham Hall, Geoff Pearson, Emma Rayner, Helen McShane, Ann Williams, Mike Dennis, Philip D. Marsh, Sally Sharpe

**Affiliations:** 1 National Infection Service, Public Health England, Porton Down, Wiltshire, SP4 0JG UK; 2 Nuffield Department of Medicine, The Jenner Institute, University of Oxford, Oxford, UK; 3 University of Cape Town, Cape Town, South Africa; 4 Churchill Hospital, Headington, Oxford, UK; 5 Colorado State University, Fort Collins, CO USA

**Keywords:** Infection, Infectious diseases, Lymphocytes

## Abstract

This study describes the use of cynomolgus macaques of Chinese origin (CCM) to evaluate the efficacy and immunogenicity of the BCG vaccine against high dose aerosol *Mycobacterium tuberculosis* challenge. Progressive disease developed in three of the unvaccinated animals within 10 weeks of challenge, whereas all six vaccinated animals controlled disease for 26 weeks. Three unvaccinated animals limited disease progression, highlighting the intrinsic ability of this macaque species to control disease in comparison to macaques of other species and genotypes. Low levels of IFNγ were induced by BCG vaccination in CCM suggesting that IFNγ alone does not provide a sufficiently sensitive biomarker of vaccination in this model. An early response after challenge, together with the natural bias towards terminal effector memory T-cell populations and the contribution of monocytes appears to enhance the ability of CCM to naturally control infection. The high dose aerosol challenge model of CCM has value for examination of the host immune system to characterise control of infection which would influence future vaccine design. Although it may not be the preferred platform for the assessment of prophylactic vaccine candidates, the model could be well suited for testing post-exposure vaccination strategies and drug evaluation studies.

## Introduction

Tuberculosis is responsible for 1.2 million deaths each year, with ten million new infections by the causative agent *Mycobacterium tuberculosis* (*M. tb*) annually^1^. Vaccination is widely accepted as the most effective method for control of infectious disease, and although the only licenced TB vaccine Bacille Calmette-Guérin (BCG) protects children from developing severe TB disease^2^, the protection afforded to adults is limited^3^, and BCG is unsuitable for use in people whose immune system is compromised. Iimproved vaccines against TB are desperately needed. Until a surrogate marker able to predict the potential efficacy of new TB vaccine candidates is identified, the slow, logistically complex and expensive process of performing large scale clinical trials involving thousands of at-risk individuals in endemic countries remains the only approach for determining vaccine efficacy^4–6^.

Animal models are valuable in the development of new vaccines, as studies of infectious challenge can be used to predict the effectiveness of vaccines in humans and provide the opportunity to identify and validate correlates of protection. Non-human primates (NHP), with their close similarity to humans, in terms of immunology and physiology provide the most relevant models of human tuberculosis^7–9^. The potential of the cynomolgus macaque species as a model for human tuberculosis following administration of *M. tb* via the trachea was first described by Walsh et al.^10^, and subsequent studies have demonstrated the propensity of the species to develop a spectrum of outcomes from active disease to chronic and latent infection^11,12^. Consequently, when using cynomolgus macaques, the number of animals required for TB vaccine evaluation experiments must be sufficient to ensure statistically robust results can be obtained in light of this inherent variability in disease control, and animals must be monitored for an extended period for the outcome of infection to be determined^12^. For these reasons, the rhesus macaque is commonly used as a stringent, but more practical model for TB vaccine evaluation due to the uniform and relatively rapid disease progression that can be observed in this species^13–16^. However, we hypothesised that a uniform profile of disease progression may be achievable in cynomolgus macaques of Chinese genotype by application of a high dose aerosol *M. tb* challenge to ensure widespread distribution of bacteria within the lung^17^, thus providing an alternate primate model for evaluation of prophylactic TB vaccination strategies.

Validated correlates of protection against *M.tb* infection are yet to be identified, although both a cell mediated T-helper 1 (Th1) response from CD4 T-cells^18^ and a MHC-I restricted CD8 T-cell response^19^ are known to be important for successful control of disease. Until a true correlate of protection is available, functional markers such as IFN-γ expression are measured to assess vaccine immunogenicity. There is evidence that the quality of the T-cell response is important to the induction of T-cell memory^20,21^ and multifunctional CD4 T-cells expressing combinations of the cytokines IFN-γ, TNF-α and IL-2 are involved in the active phase of disease^22–24^ and provide potential correlates of vaccine-induced protection^25–28^ although not all reports support this latter role^29,30^.

This study aimed to establish a high dose aerosol challenge model of TB in cynomolgus macaques of Chinese origin (CCM), in order to evaluate the efficacy afforded by BCG and to elucidate correlates of protective immunity.

## Materials and methods

### Experimental animals

Twelve 3-year-old, male cynomolgus macaques (*Macaca fascicularis*) of Chinese genotype were obtained from a Home Office approved breeding colony. Absence of previous exposure to mycobacterial antigens was confirmed by a tuberculin skin test whilst in their original breeding colony and screening using an ex-vivo IFN-γ ELISPOT (MabTech, Nacka. Sweden) to measure responses to PPD (SSI, Copenhagen, Denmark), and pooled 15-mer peptides of ESAT6 and CFP10 (Peptide Protein Research LTD, Fareham, U.K.) immediately prior to study entry as described elsewhere^16^.

Throughout the study animals were housed in compatible social groups, in accordance with the Home Office (UK) Code of Practice for the Housing and Care of Animals Used in Scientific Procedures (1989), and the National Committee for Refinement, Reduction and Replacement (NC3Rs), Guidelines on Primate Accommodation, Care and Use, August 2006 (NC3Rs, 2006). Animals were sedated by intramuscular (IM) injection with ketamine hydrochloride (Ketaset, 100 mg/ml, Fort Dodge Animal Health Ltd, Southampton, UK; 10 mg/kg) to allow procedures requiring removal from their housing as described elsewhere^16,31^. None of the animals had been used previously for experimental procedures and each socially compatible group was randomly assigned to a study treatment. All animal procedures and study design were approved by the Public Health England, Porton Down Establishment Animal Welfare and Ethical Review Body, and authorized under an appropriate UK Home Office project license and as in previous studies^16,31,32^. The study was carried out in compliance with the ARRIVE guidelines.

### Vaccination

Six macaques (Group A) were immunised intradermally (ID) in the upper left arm with 100 μl BCG vaccine, Danish strain 1331 (SSI, Copenhagen, Denmark). The method is as described elsewhere^16,32^ but in brief; BCG vaccine was prepared for ID administration according to manufacturer’s instructions for preparation of vaccine for administration to human adults, by addition of 1 ml Sautons diluent to a vial of vaccine, to give a suspension of BCG at an estimated concentration of 2 × 10^6^ to 8 × 10^6^ CFU/ml. All vaccinations were administered within one hour of vaccine reconstitution. The viability of the BCG vaccine was confirmed to be within the expected range for the batch. Vaccination sites were monitored and assessed for local reactions after vaccination with BCG.

### *M. tb* challenge strain

The stocks of the *M. tb* Erdman strain K 01 (BEI Resources) used for challenge were provided as frozen suspensions at a stated titre of 3 × 10^8^ colony forming units (CFU)/ml when grown on Middlebrook 7H11 OADC selective agar as described previously^21^. On the day of challenge, six vials were thawed, pooled and diluted appropriately, in sterile distilled water.

### Aerosol exposure

Twenty-one weeks after vaccination with BCG the six immunised animals (Group A) together with six unvaccinated animals (Group B) were challenged by the aerosol route with *M. tb.* The methodology and apparatus used to deliver *M. tb* via the aerosol route was as previously described^34^. In brief, mono-dispersed bacteria in particles were generated using a 3-jet Collison nebuliser (BGI) and, in conjunction with a modified Henderson apparatus^35^, delivered to the nares of each sedated primate via a modified veterinary anaesthesia mask. Challenge was performed on sedated animals placed within a ‘head-out’, plethysmography chamber (Buxco, Wilmington, North Carolina, USA) to enable the aerosol to be delivered simultaneously with the measurement of respired volume. The aerosol delivery process was calculated to result in the deposition of an estimated target dose of 1000 CFU in the lungs. The calculations to derive the presented dose (PD) and the retained dose (the number of organisms assumed to be retained in the lung) have been described previously for high/medium aerosol doses^36,37^. The challenge was conducted such that, one animal from each group was exposed in sequence with the cycle repeated until all animals were exposed.

Macaques were challenged by exposure to an aerosol estimated to result in a median dose of 1098 CFU (range 964–1349) deposited in the lungs, calculated by application of the retention factor described by Harper and Morton^38^. The aerosol dose was measured by air sampling during challenge, where PD = concentration of organisms in the aerosol × volume of aerosol breathed and calculated as described previously^39^. The BCG vaccinated group received a median retained dose of 1210 CFU which was not significantly different to the median dose of 1062 CFU calculated for the unvaccinated group (*p* = 0.132).

### Clinical procedures

Animals were monitored daily for behavioural and clinical changes. Behaviour was evaluated for contra-indicators including depression, withdrawal from the group, aggression, changes in feeding patterns, breathing pattern, respiration rate and cough. Prior to blood sample collection, vaccination, aerosol challenge and euthanasia, animals were weighed, examined for gross abnormalities and body temperature measured. As in previous studies^32^, red blood cell haemoglobin (RBC[Hb] levels were measured using a HaemaCue haemoglobinometer (Haemacue Ltd, Dronfield, UK) to identify the presence of anaemia, and erythrocyte sedimentation rates (ESR) were measured using the Sediplast system (Guest Medical, Edenbridge, UK) to detect and monitor inflammation induced by infection with *M. tb*. The time of necropsy, if prior to the end of the planned study period, was determined by experienced primatology staff and based on a combination of the following adverse indicators: depressed or withdrawn behaviour, abnormal respiration (dyspnoea), loss of 20% of peak post-challenge weight, ESR levels elevated above normal (> 20 mm), haemoglobin level below normal limits (< 100 g/dl), increased temperature (> 41 °C) and severely abnormal chest X-ray.

### Peripheral blood mononuclear cells (PBMC) preparation

Peripheral blood mononuclear cells (PBMC) were isolated from heparin anti-coagulated blood using standard methods^40^.

### Immunophenotyping of PBMCs

Immunophenotyping assays were applied to PBMC samples collected prior to, and following BCG vaccination or *M. tb* challenge, and basal T-cell, monocyte and NK cell population frequencies were quantified. PBMCs were thawed, washed, re-suspended in medium (R10) consisting of RPMI 1640 supplemented with l-glutamine (2 mM), penicillin (50 U/ml)/streptomycin (50 μg/ml) and 10% heat-inactivated foetal bovine serum with 1 U/ml of DNase (Sigma, UK) and incubated at 37 °C for 2 h. The antibodies used included: CD3-AF700 (FN-18) (BD Biosciences); CD4-PerCP-Cy5.5 (OKT4), CD8-APC-Fire750 (SK1), CD11c-PE (3.9), CD14-APC (M5E2), CD16-BV786 (3G8), CD20-PE-Dazzle (2H7) (all from BioLegend); CD56-BV605 (MY31), HLA-DR-BUV395 (G46-6) (both from BD Biosciences); CD159a-PC7 (Z199) (Beckman Coulter); Live/Dead Fixable Dead Cell Stain (Violet) (Invitrogen). Following staining, cells were washed and resuspended in 4% paraformaldehyde prior to collection using the BD LSRII Fortessa flow cytometer.

### Memory T-cell intracellular cytokine staining assay

Antigen specific memory T-cells secreting multiple cytokines were detected using PPD stimulated PBMCs using the method previously described (Sharpe et al*.* 2016).

### Interferon-gamma (IFN-γ) ELISpot

An IFN-γ ELISpot assay was used to estimate the numbers and IFN-γ production capacity of mycobacteria-specific T cells in PBMCs using a human/simian IFN-γ kit (MabTech, Nacka. Sweden), as described previously^34^. PBMCs were cultured with 10 μg/ml PPD (SSI, Copenhagen, Denmark), or a pool containing overlapping 15mer peptides spanning ESAT6 (Peptide Protein Research Ltd, Wickham, UK), or without antigen, in triplicate, and incubated for 18 h. Phorbol 12-myristate (Sigma-Aldrich Dorset, UK) (100 ng/ml) and ionomycin (CN Biosciences, Nottingham, UK) (1 μg/ml) were used as a positive control. After culture, spots were developed according to the manufacturer’s instructions. Spot forming units were counted and average spot areas measured using AID CADAMA ELISPOT reader and software (CADAMA, Stourbridge, UK). Determinations from triplicate tests were averaged. Data were analysed by subtracting the mean number of spots in the wells with cells in medium alone from the mean counts of spots in wells with cells combined with PPD or peptide pools.

### Quantification of secreted IFN-γ

IFN-γ production was measured in serum samples prepared from clotted blood using standard methods and supernatants collected from cultures of *M. tb* antigen stimulated whole blood^34^. Heparinised blood was diluted 1 in 10 with serum-free medium (RPMI supplemented with l-glutamine, penicillin and streptomycin (All Sigma-Aldrich, UK)) and cultured with either purified protein derivative from *M. tb* (PPD; 5 μg/ml, SSI, Copenhagen, Denmark), BCG (Danish strain 1331, 2 × 10^5^ CFU, SSI, Copenhagen, Denmark), or mitogen Phytohaemaglutinin (PHA; 10 μg/ml, Sigma-Aldrich, Dorset, UK), or in medium alone for 6 days. Supernatants were harvested at day 6 and stored at − 80 °C.

The quantity of IFN-γ in the supernatants and serum was estimated using a commercially available human/monkey IFN-γ ELISA kit (MabTech, Nacka. Sweden). A purified human IFN-γ was used for the standard curve on each plate that was included with the kit. The ELISA was developed using streptavidin-HRP and 3,3′,5,5′-tetramethylbenzidine (TMB) liquid substrate system (Sigma-Aldrich, Dorset, UK) and the reaction stopped with 2 M sulphuric acid (May & Baker Ltd, Dagenham, UK). Well absorbances at 450 nm were determined using a Multiskan reader (ThermoScientific, USA). A standard curve was plotted for each plate and used to calculate the concentrations of IFN-γ in each sample using Ascent Software (ThermoScientific, USA) to calculate the reciprocal from the standard curve. Lower limits of detection were determined on a plate by plate basis based on the standard curve. The media only wells were subtracted from the PPD or PHA stimulated sample wells.

### Whole blood intracellular cytokine staining

Immediately after collection, 500 μl undiluted, sodium heparin anticoagulated whole blood was added to tubes containing a 0.25 μg/ml solution of anti-CD28 and anti-CD49d (both from BD Biosciences, Oxford, UK), and either PPD (20 μg/ml, SSI, Copenhagen, Denmark), or nothing (negative control), or SEB (5 μg/ml Sigma Aldrich, Gillingham, UK) (positive control) and were transferred to a 37 °C water bath. After six hours, 10 μl Brefeldin-A (10 μg/ml, Sigma Aldrich, UK) was added and the water bath programmed to switch off after a further 5 h. The red blood cells were lysed using 1 ml Pharmlyse (BD Biosciences, Oxford, UK) and cells stored in cryomedia (heat inactivated foetal calf serum (Labtech International, Uckfield, UK) with 10% DMSO (Sigma Aldrich, Gillingham, UK) in nitrogen vapour.

Samples were thawed, washed, re-suspended in medium (R10) consisting of RPMI 1640 supplemented with l-glutamine (2 mM), penicillin (50 U/ml)/streptomycin (50 μg/ml) and 10% heat-inactivated foetal bovine serum with 1 U/ml of DNase (Sigma, UK) and incubated at 37 °C for 2 h. Cell concentrations were adjusted to 1 × 10^6^ cells/ml in R10. Intracellular cytokine staining to evaluate antigen-specific production of the cytokines, IFN-γ, TNF-α, and IL-2, was performed as previously described^31^.

### Flow cytometric acquisition and analysis for WBICS

Cells were analysed using a 4b SORP LSRII (BD Biosciences, Oxford, UK). Analysis was performed using an established method^21^; cytokine-secreting T cells were identified using a forward scatter-height (FSC-H) versus side scatter-area (SSC-A) dot plot to identify the lymphocyte population, to which appropriate gating strategies were applied to exclude doublet events, non-viable cells, monocytes (CD14^+^) and B cells (CD20^+^) prior to sequential gating through CD3^+^, CD8^−^ and CD4^+^ versus IFNγ, and CD3^+^, CD8^+^ and CD4^−^ versus IFNγ histograms. All data were analysed using FlowJo (version 9.7.6, Treestar, Ashland, US). Polyfunctional cells were identified using Boolean gating combinations of individual cytokine-producing CD4 or CD8 T-cells. The software package PESTLE (version 1.7) was used for background subtraction to obtain antigen-specific ICS assay responses, and SPICE (version 5.35) was used to generate graphical representations of flow cytometry data (Mario Roederer, Vaccine Research Centre, NIAID, NIH).

### Necropsy

Animals were anaesthetised, and clinical data collected. Blood samples were collected prior to euthanasia by intra-cardiac injection of a lethal dose of anaesthetic (Dolelethal, Vétoquinol UK Ltd, 140 mg/kg). A post-mortem examination was performed immediately, and gross pathological changes were scored using an established system based on the number and extent of lesions present in the lungs, spleen, liver, kidney and lymph nodes, as described previously^34^. Samples of spleen, liver, kidneys and tracheobronchial, inguinal and axillary lymph nodes were removed and sampled for quantitative bacteriology. The lungs, including the heart and attached tracheobronchial and associated lymph nodes, were removed intact. The lymph nodes were measured and examined for lesions. Following examination, the left bronchus of lung was clamped off using artery forceps, and the left upper and left lower lung lobes dissected away from lung. The upper and lower left lobes were collected for quantitative bacteriology. The left bronchus was tied off with string to the left of the clamp and right-hand side of lung gently infused with 10% neutral buffered formalin (NBF) using a 10 ml syringe attached to a 14 CH Netalon catheter (J.A.K. Marketing, York, UK) whilst observing closely for inflation. The trachea was tied off and the lungs immersed in formalin to complete fixation. In addition, samples of kidneys, liver, spleen, and sub clavicular, hepatic inguinal and axillary lymph nodes were fixed in 10% NBF.

### Lung imaging

Thoracic radiographs (SP VET 3.2, Xograph Ltd) were acquired using mammography film (Xograph Imaging Systems Ltd, Tetbury, UK) before and every 2 weeks after exposure to *M. tb*. Evaluation of disease was performed by an experienced consultant thoracic radiologist blinded to the animal group and clinical status, using a pre-determined scoring system based on the amount and distribution of infiltrate^39^. The lung was divided into four zones (left and right sides divided at the anterior aspect of the fifth rib) and the disease burden scored in each area according to the system 0 = normal; 1 ≤ 10% abnormal; 2 ≥ 10% < 50% abnormal; 3 ≥ 50% < 75% abnormal; 4 ≥ 75% abnormal. The scores for each zone were summed to provide the total X-ray score where the maximum score was 16.

### Magnetic resonance imaging

The ex-vivo expanded, fixed lungs were set in 2% agarose (Sigma-Aldrich, UK) and images were taken using a 3.0 T 750 MRI Scanner (General Electric Healthcare, Milwaukee, WI, USA) as described previously^34^. This enabled evaluation of the pulmonary disease burden at the end of the study period. Lung lesions were identified in MR images from their signal intensity and nodular morphology relative to normal lung parenchyma.

### Lesion analysis/quantification (stereology)

Lung lesions were identified on MR images based on their signal intensity and nodular morphology relative to normal lung parenchyma. The total lung and lesion volume relative to the fixed tissue was determined using the Cavalieri method applied to MRI image stacks, and then expressed as a ratio to provide a measure of disease burden in each animal as previously described^34,39^. Analyses of lesion volume on magnetic resonance (MR) images were performed with the investigators reading the images blind to treatment groups.

### Histopathological examination

Representative samples from each lung lobe and other organs, were processed to paraffin wax, sectioned at 3–5 µm and stained with haematoxylin and eosin (HE). For each lung lobe, tissue slices containing obvious lesions were selected for histological examination. Where gross lesions were not visible, a sample was taken from a pre-defined anatomical location from each lobe to establish consistency between animals. Sections of lung associated lymph nodes (trachea-bronchial at the bifurcation and cranial and caudal to the bifurcation) and other tissues were evaluated for the presence of tuberculous lesions. Lesions were classified according to the scheme used by Lin et al.^12^.

### Bacteriology

The lung lobes, spleen, kidneys, liver and tracheobronchial lymph nodes were sampled for the presence of viable *M. tb* post-mortem as described previously^34^. Weighed tissue samples were homogenized in 2 ml (spleen, liver, kidney, hilar lymph nodes) or 10 ml (lung lobes) of sterile water, then either serially diluted in sterile water prior to being plated or plated directly onto Middlebrook 7H11 OADC selective agar. Plates were incubated for 3 weeks at 37 °C and resultant colonies were confirmed as *M. tb* and counted.

### IDEXX Lasercyte haematology analyser

The IDEXX Lasercyte analyser (IDEXX, USA) was used according to the manufacturer’s instructions using 500 µl of whole blood anticoagulated with EDTA (1.8 mg/ml of blood), in a Vacutainer (BD Biosciences, USA).

### Enzyme-linked immunosorbent assay (ELISA)

ELISAs were performed as previously described^41^. Antigens used were purified protein derivative (PPD) (Statens Serum Institut (SSI), Denmark) at a concentration of 5 µl/ml and whole BCG SSI at a concentration of 5 × 10^5^ CFU/ml; plates were coated with 50 µl of antigen and incubated at 4 °C overnight. Samples were prepared by diluting test serum and positive/negative control serum 1:100 in casein and 50 µl added per well for 2 h. Secondary antibody (goat anti-monkey γ-chain-specific whole IgG alkaline phosphatase conjugate (Rockland Immunochemicals, PA, US) was diluted 1:750 and 50 µl added per well for 1 h. 100 µl of p-nitrophenyl phosphate (pNPP) development buffer was added to each well and plates were read every 10 min using a Model 550 Microplate Reader (Bio-Rad, UK) until the positive control reached a predetermined OD_405_ that was consistent across plates. Reported values are the mean OD_405_ values of triplicate samples with the mean OD_405_ values of triplicate negative controls subtracted.

### Mycobacterial growth inhibition assay

The direct PBMC mycobacterial growth inhibition assay (MGIA) was carried out using PBMCs at a concentration of 13 × 10^6^ cells inoculated with ~ 500 CFU Pasteur Aeras in a total volume of 480 µl RPMI (containing 2 mM l-glutamine and 25 mM HEPES), plus 120 µl autologous serum matched to animal and time-point in a 48-well plate, as described previously^42^. After incubation at 37 °C with 5% CO_2_ for 96 h, co-cultures were transferred to 2 ml screw-cap tubes and centrifuged at 15,300×*g* for 10 min. During this time, 500 µl sterile water was added to each well to lyse adherent monocytes and release intracellular mycobacteria. Supernatants were removed from the screw-cap tubes by pipetting, and water from the corresponding well added to the remaining pellet. Tubes were pulse vortexed and the suspension was transferred to Mycobacterium growth indicator tubes (MGIT) supplemented with PANTA antibiotics and OADC (Becton Dickinson, UK) for enumeration of surviving mycobacteria using the BACTEC MGIT instrument (Becton Dickinson, UK). On day 0, duplicate direct-to-MGIT inoculum controls were set up by inoculating supplemented BACTEC MGIT tubes with the same number of mycobacteria as the samples. The time to positivity (TTP) read-out was converted to log10 CFU using stock standard curves of TTP against inoculum volume and CFU. Results were normalised by subtracting log10 CFU of the inoculum control from log10 CFU of each sample, and ‘vaccine response’ calculated as (post-vaccination normalised growth–baseline normalised growth), presented as Δlog10 CFU.

### Statistical analyses

Comparisons of ex-vivo IFNγ ELISpot profiles were completed using the area under the curve (AUC) of each animal’s response calculated using Sigmaplot version 10 (Systat Software Inc, Hounslow, UK). AUC values were compared between test groups using the non-parametric Mann–Whitney U test, Minitab version 15 (Minitab Ltd, Coventry, UK).

To compare T-cell functional profiles measured by polyfunctional flow cytometry, between vaccination groups, T-cell subset frequencies were compared using a Wilcoxon-rank test at each analysis time point (SPICE v 5.35). Similarly, vaccine-induced changes in T-cell functional profiles within each vaccination group were assessed by comparing frequencies at each analysis time point with mean baseline values. Negative values in antigen-specific ICS data generated by background subtraction were replaced by a minimum threshold value^43^*.*

Pathology scores, pulmonary disease measures and clinical measures of disease burden at the end of study were compared between test groups using the non-parametric Mann–Whitney U test, Minitab version 15 (Minitab Ltd, Coventry, UK). The ability to control disease progression by animals in each test group were compared with a log rank test using Minitab version 15 and Gehan–Breslow–Wilcoxon test in Graphpad Prism, version 5.01 (GraphPad Software Inc, La Jolla, California, USA).

The Spearman correlation test was used to determine the level of correlation between study parameters using GraphPad Prism, version 5.01 (GraphPad Software Inc, La Jolla, California, USA).

### Ethics declaration

All animal procedures and study design were approved by the Public Health England, Animal Welfare and Ethical Review Body, Porton Down, UK, and authorised under an appropriate UK Home Office project license.

## Results

### Disease progression

**Figure 1:**
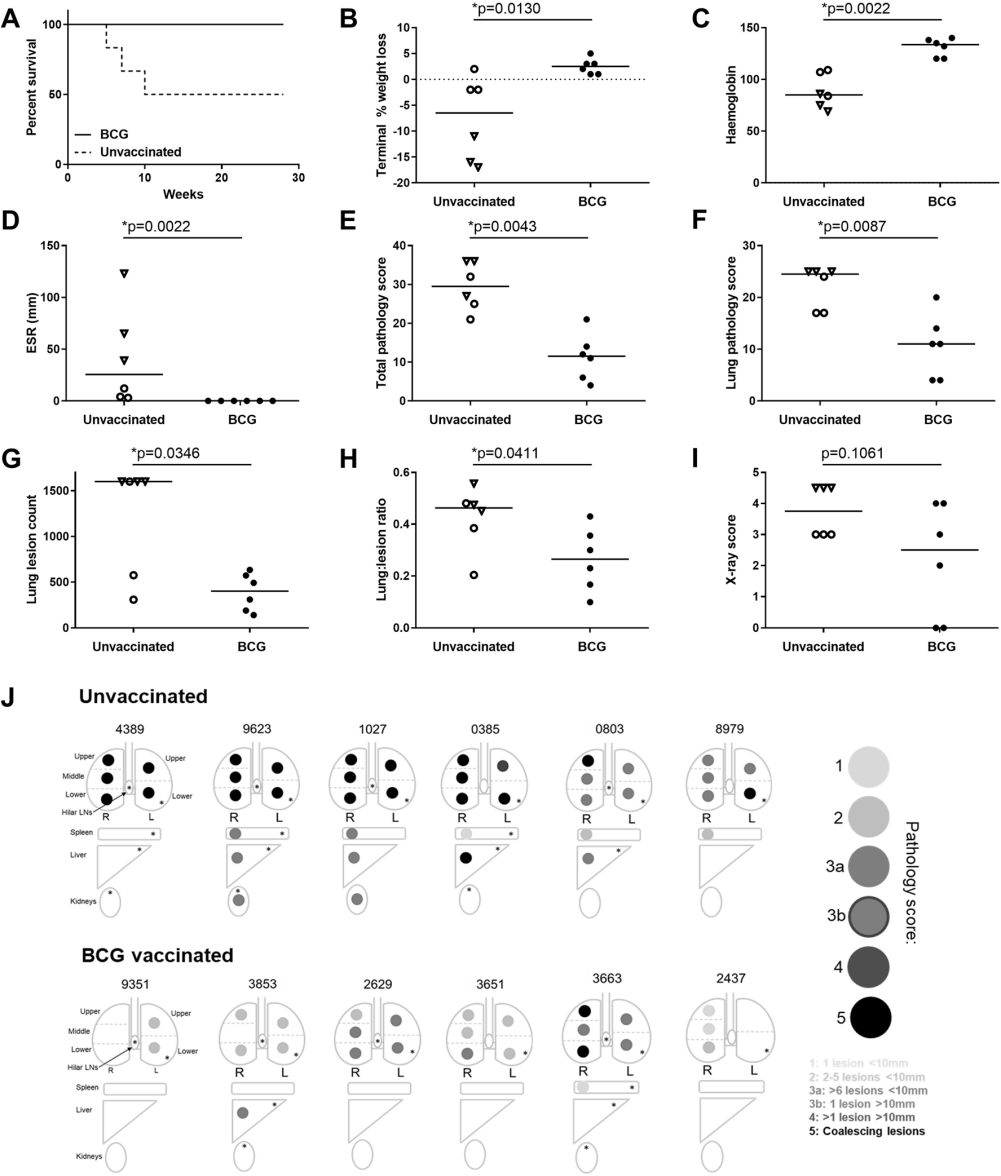
Measures of tuberculosis-induced pulmonary and clinical disease burden. (**A**) Kaplan Meier plot of survival of BCG vaccinated and unvaccinated NHPs after challenge with *M. tb*, (% weight loss from peak post-challenge weight on the day of euthanasia, (**C**) Red cell haemoglobin concentration on the day of euthanasia, (**D**) Erythrocyte sedimentation rate (ESR) on the day of euthanasia, (**E**) the total pathology score determined using a qualitative scoring system, (**F**) the score attributed to the pulmonary component as part of the total pathology; (**G**) number of lesions in the lung enumerated by serial sectioning and manual counting, (**H**) the lung to lesion volume ratio determined using MR stereology, (**I**) score attributed to the chest radiogram on the day of euthanasia. Graphs B-I medians shown. Black symbols = BCG vaccinated, open symbols = unvaccinated. Triangle symbols indicate animals in which disease reached levels that met endpoint criteria. p values are shown and *denotes significant difference in outcome between groups using the non-parametric Mann–Whitney U test, p ≤ 0.05. (**J**) representative diagram of the location and number of granulomas present in the lungs, hilar lymph nodes, spleen, liver and kidneys and *show where *M. tb* bacteria were isolated and cultured from.

In the period after aerosol challenge with *M. tb*, three (4389, 9623, 1027) of the six animals that did not receive a BCG vaccination showed changes in behaviour and clinical parameters consistent with progression of tuberculosis (decreased body weight, anaemia, raised inflammatory markers, severely pulmonary changes) and were euthanized ahead of the planned end of the study at 5, 7 and 10 weeks after challenge. By contrast, all six BCG vaccinated animals controlled disease progression during the 26 weeks of study (Fig. 1A). The length of time that animals in each group controlled disease progression was compared using two statistical approaches, the Log Rank Test and Cox regression analysis. Both tests demonstrated trends for improved control of disease progression in the BCG vaccinated group although neither reached statistical significance (Log rank test, *p* = 0.055, Cox regression, *p* = 0.058).

Trends for body weight to increase over time and body temperature to remain within normal ranges were seen in both the BCG vaccinated and unvaccinated animals that completed the study period. At the end of the study significantly more weight loss was recorded in the unvaccinated group than the BCG vaccinated group (*p* = 0.0130, Fig. 1B). The BCG vaccinated group showed (RBC[Hb]) levels consistently within the normal range for the species, while the levels in the unvaccinated animals reduced over time and tended to be low at termination in the animals in which disease progressed to meet humane end criteria. Erythrocyte sedimentation rate (ESR) increased in animals as disease progressed to meet humane endpoint criteria but remained within the normal range in all animals that completed the study. At the end of the study, RBC[Hb] levels were significantly higher (*p* = 0.0022, Fig. 1C) and ESR significantly lower (*p* = 0.0022, Fig. 1D) in the BCG vaccinated group than the unvaccinated group.

Disease burden was evaluated, either at the planned study endpoint (26 weeks after aerosol exposure), or when disease progressed to meet pre-set, humane, endpoint criteria. At necropsy, all animals were assessed for gross pathological changes. All BCG vaccinated animals were seen to have good body condition and the abdominal organs were free of observable abnormalities. In the thoracic cavity scattered lesions were seen on the lung surfaces of all six animals with enlarged lymph nodes observed in two animals. In contrast, two of the six unvaccinated animals showed thin body condition, and abnormalities were detected in the gut or abdominal region of three of the six animals (for example; enlarged gall bladder or spleen, pale liver colour) and all six animals possessed abnormalities in the thoracic cavity (e.g. adhesions to thorax wall). TB-induced disease burden was higher in animals in which disease met humane endpoint criteria during the study, than in those which controlled disease progression to the end of the study. The total pathology score determined at necropsy, based on the number and size of lesions, revealed the level of disease in all BCG vaccinated animals to be significantly lower than in the unvaccinated animals (*p* = 0.0013, Fig. 1E).

The pathology and imaging scores used to assess pulmonary disease revealed differences in outcome between the vaccinated and unvaccinated groups. The lung pathology scores (Fig. 1F), comprising the number of pulmonary lesions determined by serial sectioning and manual counting of discrete and coalesced lesions in the right lung lobes (Fig. 1G), and pulmonary disease burden quantified using stereology of MR images to determine the ratio between lesion volume and lung volume in the left lung lobes (Fig. 1H), were significantly lower in the BCG vaccinated group (lesion count: *p* = 0.0346; Lung pathology score: *p* = 0.0087*;* lesion:lung volume: *p* = 0.0411) than in the unvaccinated group. Chest X-ray scores also revealed a non-significant trend for a reduced level of pulmonary disease in the BCG vaccinated animals (*p* = 0.106, Fig. 1I).

Macroscopic assessment of the lungs revealed tuberculous lesions in all animals in both study groups with a tendency for disease to be more severe in the unvaccinated group (Supplementary Data 1), which was confirmed by microscopic assessment. A range of granuloma types with variable presence of mineralisation and coalescing lesions were seen in the lungs of both BCG vaccinated and unvaccinated animals. Unorganised granulomas were observed more frequently in BCG vaccinated animals than in the unvaccinated animals, and coalesced lesions were observed more frequently in the unvaccinated animals particularly those which developed progressive disease during the study.

Evidence of dissemination to the hilar lymph nodes, liver, kidneys and spleen was sought by gross and microscopic pathological examination and by culture of tissue samples for the presence of viable *M. tb* (Fig. 1J)*.* Tuberculous lesions were observed in the hilar lymph nodes of all study animals and *M. tb* was isolated from the hilar lymph nodes of four of six animals in each group (Supplementary Fig. S2). Spread to the thoracic walls was seen only in the unvaccinated animals where tuberculosis lesions were grossly visible in four of the six animals. In the BCG vaccinated animals, gross lesions were not observed in any abdominal organs but microscopic examination and bacterial culture, suggested a trend for reduced spread to the spleen (three of six), liver (three of six) and kidneys (three of six) in the BCG vaccinated animals compared to the unvaccinated animals (spleen: five of six; liver: five of six; kidneys four of six). (Fig. 1J, Supplementary Fig. S2). Tuberculous lesions were not detected in the brain of animals from either group. Where bacteria were isolated, the bacterial burden in tissues was at a similar level in both study groups (Supplementary Fig. S2). Mycobacteria were not isolated from the urine, faeces, saliva or PBMCs of any of the vaccinated or unvaccinated animals.

### The mycobacterium specific IFNγ response to BCG vaccination and challenge with *M. tb*

IFNy is an important biomarker for TB, consequently a series of approaches were used to characterize the IFNγ response induced by BCG vaccination and *M. tb* infection in CCM.

**Figure 2:**
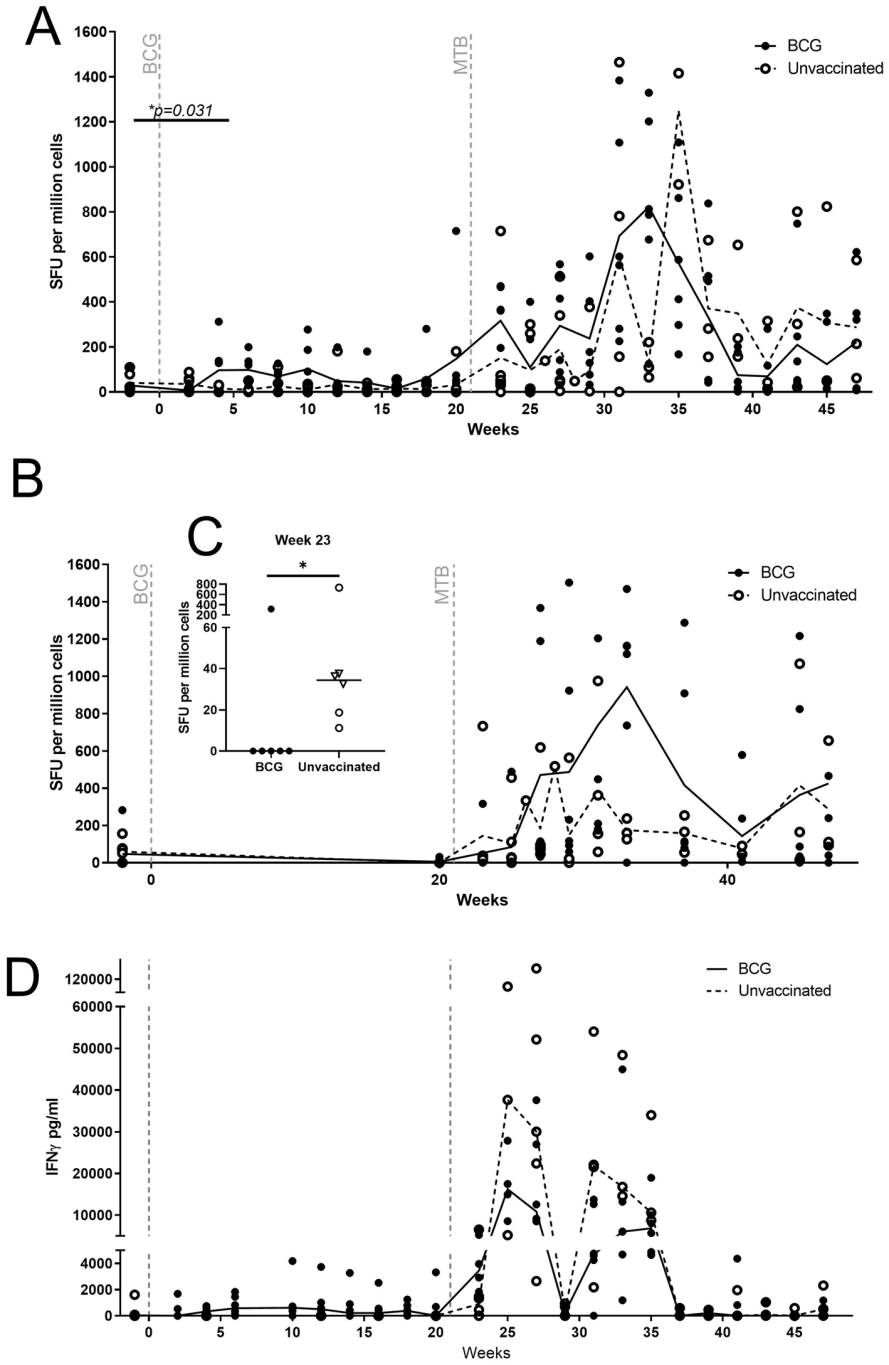
IFNγ detected using ELISPOT and ELISA in CCM with and without BCG vaccination and challenged with *M. tb* at week 21. (**A**) IFNγ ELISPOT PPD-specific SFU per million cells, (**B**) IFNγ ELISPOT ESAT-6-specific SFU per million cells, (**C**) IFNγ detected using whole blood ELISA, PPD-specific. CCM were either vaccinated with BCG (n = 6) or unvaccinated controls (n = 6 to week 25, n = 5 to week 27, n = 4 to week 29 then n = 3 to week 47). Lines indicate median and dots represent individual animals. Solid line = BCG vaccinated, hashed line = unvaccinated macaques.

Firstly, application of an ex-vivo ELISPOT assay prior to and after vaccination revealed the frequency of PPD-specific IFNγ secreting cells increased to levels significantly above pre-vaccination levels in BCG vaccinated animals 6 weeks after vaccination (*p* = 0.031) (Fig. 2A) and when the AUC of the IFNγ profile was compared, a trend for the responses made by the BCG vaccinated group to be higher than those of the unvaccinated group (*p* = 0.065) was seen (Supplementary Data 3A). Challenge with *M. tb* induced an increase in the frequency of PPD-specific IFNγ secreting cells in both the vaccinated and unvaccinated animals. In the first 4 weeks after challenge, a higher PPD-specific response was observed in the BCG vaccinated group than in the unvaccinated group, although statistical analysis showed the difference in frequency at two (*p* = 0.132) and AUC up to 4 weeks after challenge did not reach significance (*p* = 0.18) (Supplementary Data 3B,C). A significant increase in the number of PPD-specific IFNγ secreting cells was observed in the BCG group 2 weeks after challenge compared to pre-challenge numbers (*p* = 0.0313). Twelve weeks after challenge there was a trend for the frequency of PPD-specific IFNγ secreting cells to be significantly higher in BCG vaccinated animals than in the three unvaccinated animals that controlled disease progression (*p* = 0.048) (Supplementary Data 3D). Fourteen weeks post-challenge (week 35) there was a transient increase in PPD-specific IFNγ secreting cells measured in the unvaccinated group, which decreased and then remained stable for the rest of the study.

The ex-vivo ELISPOT assay was applied to evaluate the response to ESAT6, a TB specific marker associated with disease burden that is absent in BCG (Fig. 2B). ESAT6-specific IFNγ-secreting cells were not detected in any of the animals prior to, or after vaccination. Following exposure to *M. tb*, significantly more ESAT6-specific IFNγ-secreting cells were measured in the unvaccinated animals than in the BCG vaccinated group 2 weeks after challenge (*p* = 0.0346); the number of spot forming units (SFU) was very low (median of 34 SFU) (Fig. 2C). The frequency of ESAT6-specific IFNγ secreting cells increased in the peripheral blood of all BCG vaccinated and unvaccinated animals with the two groups showing a similar magnitude of responses measured at four (Mann Whitney: *p* = 0.178), and 12 weeks (Mann Whitney: *p* = 0.167) after challenge and over the 26 weeks post challenge period (AUC, Mann Whitney: *p* = 0.619).

An ELISA was used to measure f IFNγ secreted in response to stimulation with PPD by cells in whole blood (WB) samples collected following vaccination and challenge, (Fig. 2D). Responses to PPD were detected in five of the BCG vaccinated animals but this was not significantly different to the unvaccinated group (*p* = 0.0827). Following exposure to *M. tb,* the PPD-specific responses induced in both the BCG-vaccinated and unvaccinated groups showed a bi-phasic pattern with peak IFNγ secretion detected between weeks 4 and 6 and weeks 10 and 12 post challenge with non-significant trends for responses to be higher in the unvaccinated group (PPD: *p* = 0.329).

The Spearman’s Rank correlation test was applied to evaluate potential relationships between the IFNγ responses induced by vaccination, or challenge and the TB-induced disease burden evaluated at termination using a range of measures, including gross pathology score, pulmonary lesion counts, lesion to lung volume ratio and changes in clinical parameters. When results from BCG vaccinated and unvaccinated animals were combined and tested, a higher frequency of PPD-specific IFNγ secreting cells measured 2 weeks after challenge correlated with lower disease burden in the lungs after challenge (week 2: lung lesion ratio: *p* = 0.013, lung score: *p* = 0.049*,* total score *p* = 0.0075) (Supplementary Data 4)*.* Significant relationships between IFNγ measures and measures of protection were not identified within individual test groups.

### Polyfunctional and memory T-cell analysis

**Figure 3:**
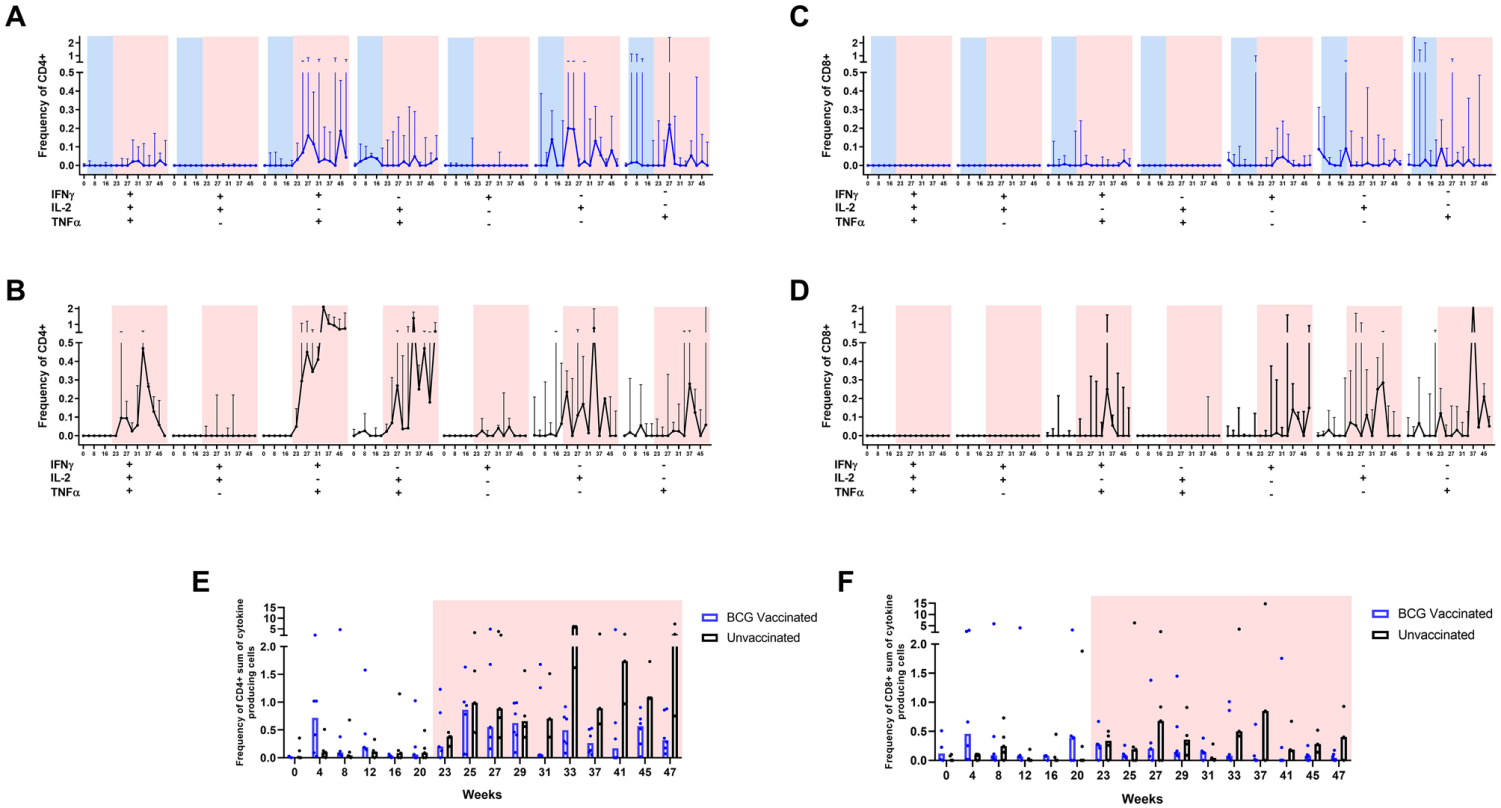
PPD-specific polyfunctional T-cell analysis of CCM with or without BCG vaccination and challenged with MTB at week 21. (**A**) CD4^+^ T-cells: vaccinated with BCG (n = 6), (**B**) CD4^+^ T-cells unvaccinated (n = 6 to week 25, n = 5 to week 27, n = 4 to week 29 then n = 3 to week 47). (**C**) CD8^+^ T-cells, vaccinated with BCG (n = 6). (**D**) CD8^+^ T-cells unvaccinated (n = 6 to week 25, n = 5 to week 27, n = 4 to week 29 then n = 3 to week 47). (**E**) Summed cytokine responses in CD4^+^ T-cells, (**F**) summed cytokine responses in CD8^+^ T-cells. Median baseline response determined from assays applied on three separate occasions prior to vaccination. Vaccination phase in blue, challenge phase in red. Interquartile range shown and black line indicates median response. Black dots show individual responses. Frequency of parent combinations of IFNγ, IL-2 and TNFα. Threshold of 0.05 (75% confidence interval). Asterisks denote significant differences between the vaccinated group and controls animals determined by Mann–Whitney test (*p ≤ 0.05).

The frequency of polyfunctional CD4^+^ and CD8^+^ T-cells producing IFNγ, TNFα and IL-2 in response to PPD stimulation of whole blood was measured both the immunisation and challenge phases of the study. Low levels of CD4+ T-cells producing IFNγ^+^ and TNFα^+^ or IL-2^+^ and TNFα^+^ were detected after vaccination, when compared to frequencies measured before vaccination, or in the unvaccinated group (Fig. 3A,B), although these did not reach significance.

Following *M. tb* challenge, increases in the frequency of CD4 T-cell populations producing IFNγ^+^ TNFα^+^ IL-2^+^, IFNγ^+^ TNFα^+^ or IL-2^+^ TNFα^+^ were detected in both vaccinated and unvaccinated animals, with higher proportions of these populations occurring in the unvaccinated group.

Minimal levels of cytokine production were detected in CD8^+^ T-cells after vaccination, though TNFα producing cells were detected in some of the animals (Fig. 3C,D) independent of vaccination status. Post-challenge, IFNγ^+^ TNFα^+^ double positive cells were detected in both vaccinated and unvaccinated animals, as well as cells producing IFNγ^+^, TNFα^+^ and IL2^+^ individually. All cytokines showed trends towards higher levels in the unvaccinated group than in BCG vaccinated animals.

When cytokine responses were summed, a significantly higher level of cytokine production was detected in CD4^+^ T-cells collected 4 weeks post-BCG vaccination compared to that in the unvaccinated group (*p* = 0.0476) (Fig. 3E). In general, higher levels of cytokine production were observed in CD8^+^ T-cells from BCG vaccinated animals during the vaccination period (Fig. 3F). Overall, summed cytokine levels measured in CD4^+^ T-cells were not significantly different in vaccinated and unvaccinated groups during the first 8 weeks post-challenge, and for the latter part of the study, higher levels of cytokine production were detected in the unvaccinated animals.

Flow cytometric analysis of PBMCs collected 20 weeks after vaccination, collected to assess the proportions of central memory T-cells (TCM), transitional memory T-cells (TEM1) and terminal memory T-cells (TEM2) that were present in the week prior to TB infection. These cells were collected to determine whether the profile of effector and memory T-cell subsets induced by BCG vaccination could be a factor in the protection seen in this group post-infection. No difference between the vaccinated, or unvaccinated groups in terms of fold change in memory populations of CD4^+^, or CD8^+^ T-cells, were seen between baseline and week 20 post-vaccination (Supplementary Data S5). The assay was also used to identify antigen-specific memory populations via the detection of cytokines, although measurable cytokine was not detected by this assay at this time point.

### Non-antigen specific immune changes, humoral immunity and in vitro bacterial growth inhibition

Further analyses including the quantification of IFNγ and mycobacteria-specific IgG titres in serum, monocyte:lymphocyte ratio (M: L), characterisation of monocyte cell subsets by immunophenotyping, and the assessment of mycobacterial growth inhibition (MGIA) were applied to identify potential biomarkers induced by BCG vaccination, or *M. tb* infection.

**Figure 4:**
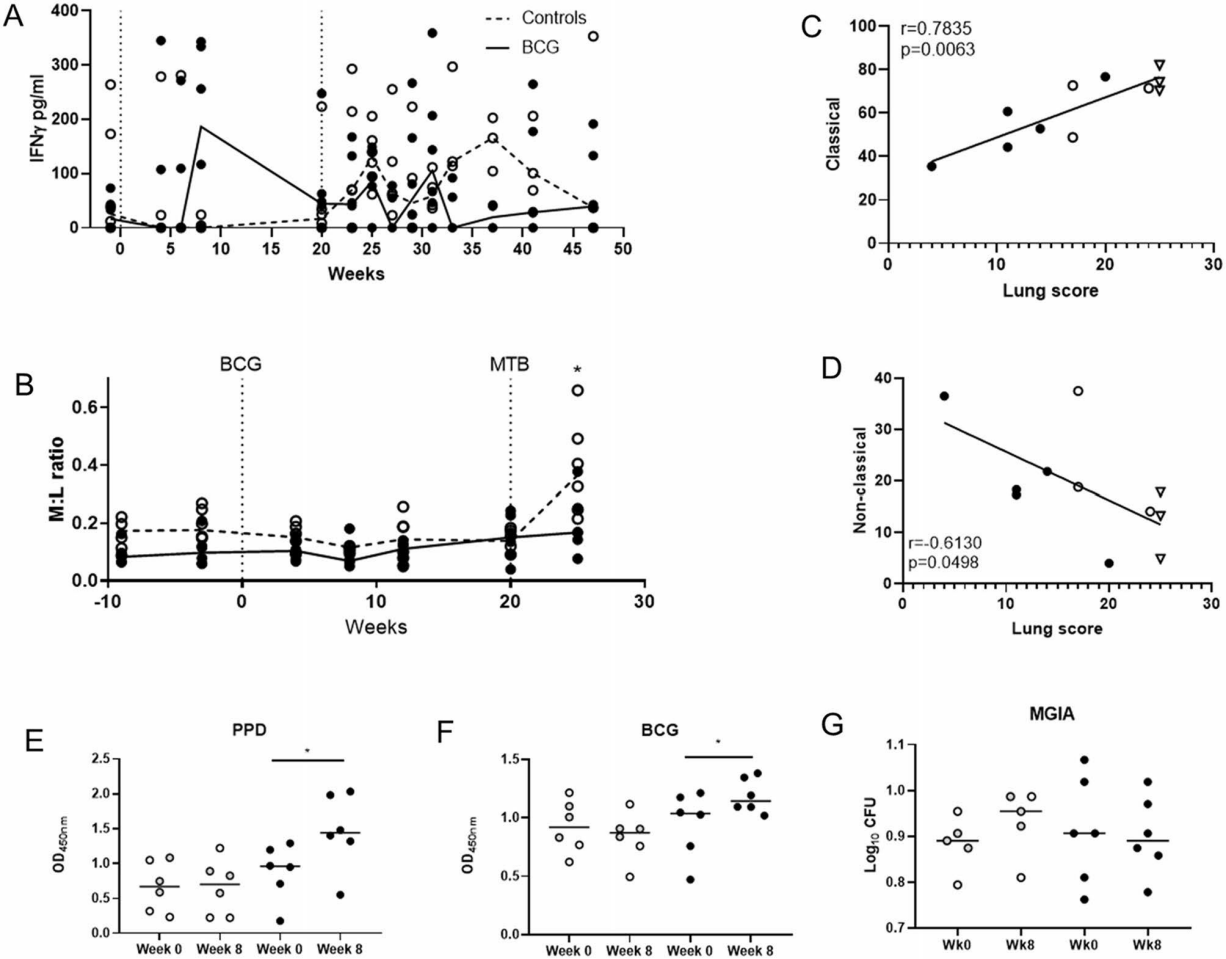
IFNγ detected in serum samples, Monocyte:lymphocyte ratio, monocyte subset analysis, analysis and levels of antigen-specific IgG and MGIA assay results. (**A**) IFNγ detected in serum using ELISA, (**B**) M:L ratio across the time course, (**C**) correlation of CD14^+^ monocytes and lung pathology score, (**D**) correlation of CD16^+^ monocytes and lung pathology, (**E**) PPD-specific IgG detected by ELISA in naïve and vaccinated CCM at 8 weeks post-vaccination measured by optical density (OD). (**F**) BCG-specific IgG detected by ELISA in naïve and vaccinated CCM at 8 weeks post-vaccination measured by optical density (OD). (**G**) MGIA assay at 8 weeks post-vaccination. Wilcoxon matched-pairs test (*p* = 0.05). White = unvaccinated, black = BCG vaccinated. Mann–Whitney t-test carried out at each time point,

An IFNγ ELISA was used to measure the quantity of IFNγ in serum and revealed an increase in the levels of IFNγ measured following vaccination in five of the six BCG vaccinated animals relative to the unvaccinated group although the difference did not reach statistical significance (p = 0.179). This trend reversed following challenge when higher levels of IFNγ were detected in the unvaccinated animals (Fig. 4A).

Using a haematology analyser, populations of neutrophils, monocytes, lymphocytes, eosinophils and basophils were measured throughout the vaccination phase and up to the fourth week after infection (Supplementary Data 6). Significantly more lymphocytes were detected in the BCG vaccinated group 4 weeks post-infection (Supplementary Data 6). The M:L ratio was calculated and whilst significant differences were not seen between the vaccinated and unvaccinated animals during the vaccination phase, significantly higher M:L ratios were recorded in the unvaccinated group in comparison to the BCG vaccinated animals 4 weeks after infection (Fig. 4B). Furthermore, the M:L ratio calculated at baseline correlated with total pathology score after infection (*r* = 0.6245*, p* = 0.033) (Supplementary Data 7).

Due to the trend seen in the M:L ratio data, monocytes were examined in more detail at baseline using a flow cytometric immunophenotyping assay (Supplementary Data 8). Monocyte subtypes were analysed using CD14 and CD16, to elucidate classical (CD14^+^ CD16^−^), intermediate (CD14^+^ CD16^+^) and non-classical (CD14^−^ CD16^+^) monocytes. When correlated with lung pathology, there was a significant positive correlation between classical monocytes and lung pathology, and an inverse trend with the non-classical monocytes (Fig. 4C,D).

The quantity of IgG specific to either PPD (Fig. 4E), or BCG (Fig. 4F) was evaluated by ELISA in serum collected at baseline and 8 weeks after vaccination to determine whether specific antibodies were induced by BCG vaccination. A significant increase in both PPD and BCG specific-IgG was detected at week 8 post-vaccination in the BCG group using a paired Wilcoxon T-test (*p* = 0.0313 for both) with no change in the unvaccinated group.

The direct mycobacterial growth inhibition (MGIA) assay was carried out using PBMCs and matched serum collected at baseline and week 8 post-vaccination. Control of mycobacterial growth was observed in three of the six BCG vaccinated animals following vaccination, and none of the unvaccinated animals (Fig. 4G). There were no significant associations between MGIA outcome and measures of protection, although there was a trend towards a correlation between mycobacterial growth at week 8 post-vaccination and X-ray score (*r* = 0.55*, p* = 0.07).

## Discussion

In this high dose infection model, BCG vaccination afforded a consistent and noteworthy level of protection, with all vaccinated animals showing a significantly reduced disease as determined by every measure of pulmonary disease, namely; lesion counts, stereology and total pathology score; overall disease burden (total gross pathology score); disease dissemination (histology, bacterial culture) and changes in clinical parameters (body weight, anaemia (RBC[Hb]) and inflammatory markers (ESR). The strong consistent protective effect conferred by BCG vaccination in CCM against high dose aerosol challenge in the present study is consistent with the protective effect afforded by BCG to similar animals against bronchoscopically delivered high dose *M. tb* challenge^44^. A feature of aerosol delivered challenge is that the inoculum is more evenly distributed throughout the lungs^17^. Thus, we aimed to use this feature to minimise variation in disease readouts and end-point measures by standardising the disease progression with a post challenge follow up period that is more amenable to vaccine evaluation than might be required following lower dose exposure in this species^12,45^.

Recent studies have used low doses of *M. tb* for intrabronchial challenge in CCM to provide a dose that is more representative of natural infection and under these circumstances the CCM shows a spectrum of outcomes, from the development of active disease through to the development of latent disease^12^. Whilst this reflects the outcome of *M. tb* infection in humans, for this approach to be used for the evaluation of vaccine efficacy, test group sizes need to be sufficient to discriminate a vaccine effect against the background of inter-animal variability. The remarkable ability of the CCM to naturally control *M. tb* infection, even after challenge with high titres of *M. tb,* is highlighted by the outcome observed in unvaccinated animals in the present study, where half successfully controlled disease for up to 6-months, illustrating the challenge of optimal group size selection for vaccine evaluation in this species. The enhanced natural ability to control *M. tb*-induced disease may also play a role in the strong protective efficacy afforded by BCG vaccination to cynomolgus macaques that is in contrast to the lower level of protection conferred by BCG to rhesus macaques^32,34^. Therefore, the natural resistance to TB of CCM populations is likely to confound efficacy measures when this species is used for the evaluation of prophylactic vaccines; however, this same feature is a useful attribute for the study of latent or chronic disease states.

As the mycobacterium-specific IFNγ response provides a biomarker often used in clinical trials^6,46–48^ and TB diagnostics^49^, the IFNγ response to vaccination and high dose aerosol challenge in the CCM was measured using several complimentary detection methods. Low levels of IFNγ were induced by BCG vaccination in CCM, which contrasts with the significantly increased IFNγ responses measured in similarly treated rhesus macaques^32,34^ and suggests that IFNγ alone does not provide a sufficiently sensitive biomarker of vaccination in this model.

IFNγ SFU as measured by ELISPOT increased after exposure to *M. tb* in both vaccinated and unvaccinated groups. The frequency of PPD-specific IFNγ producing cells increased more rapidly in BCG vaccinated animals during the initial phase after challenge with an early peak in response measured 2 weeks after challenge. In comparison, the PPD-specific IFNγ responses reported in similarly vaccinated and challenged rhesus macaques typically occurred 4 weeks after challenge^21,34^. This rapid onset of the adaptive cellular immune response in the CCM may be a factor in the improved protection conferred against challenge and is in keeping with similar observations of early increases in pro-inflammatory cytokines reported by Dijkman et al. in comparison to rhesus macaques^50^.

Following TB infection, populations of multifunctional CD4^+^ T cells increased in both vaccinated and unvaccinated groups of cynomolgus macaques, with a trend for frequencies of these cell populations to be greater in the unvaccinated group in which the highest pathology scores were recorded. This supports the hypothesis that high numbers of multifunctional CD4^+^ T-cells are related to active disease^22^. However, within the unvaccinated group, animals that were able to control disease to the end of the study had the highest levels of multifunctional cells in comparison to animals in which disease progressed to meet end-point criteria. Therefore, it appears that polyfunctional T-cells contributed to disease control for unvaccinated animals, and that polyfunctional T-cells are a central component of the mycobacterial response but are not necessarily a correlate of protection.

There appears to be a much larger population of terminally differentiated memory (TEM2) cells in CCM in comparison to rhesus macaques, where memory T-cell populations were more biased towards transitional effector memory cells in naïve animals^21,32^. These terminally differentiated memory cells are considered to be a short lived population capable of exerting a rapid but more limited range of functional parameters^20^. TEM2 cells have recently been shown to be promoted following vaccination with cytomegalovirus (CMV) vectored TB vaccines and were associated with the improved protective efficacy imparted to rhesus macaques following *M. tb* infection^15^. The natural bias towards TEM2 populations we have observed in CCM in this study and the early responses after infection may contribute to the inherent ability of this species to control TB.

M:L ratio and monocyte subtype at baseline correlated with pathology scores, and a role for the innate M:L ratio and other host factors in determining the risk of developing active disease may exist. In our previous work, we have observed that CCM have a significantly lower M:L ratio than rhesus macaques and Mauritian cynomolgus macaques^51^ and are better at controlling TB disease^33,50,52^, which corresponds to human data suggesting that high M:L ratio is a risk factor for TB^53,54^. Differences in monocyte sub populations between rhesus and CCM have been reported elsewhere; higher levels of mycobacterial permissive monocytes were found in rhesus macaques and were thought to have a negative effect on T-cell responses and have a role in the susceptibility of this species to TB^50^. Specific cell populations have been identified that may be implicated, namely; the TEM2 T-cell phenotype with a low threshold of activation, and pro-inflammatory monocyte subsets. Further work is required to elucidate whether these populations act in a coordinated manner or more independently to bring about an early response that aids disease control.

In summary, the immune response to BCG and *M. tb* in the CCM model is complex, an early response and contribution of monocytes appears to enhance the ability of CCM to naturally control infection in comparison to rhesus macaques. The high dose aerosol challenge model in CCM has value for examination of the host immune system to characterise control of infection which would influence future vaccine design, although it may not be the preferred platform for the assessment of prophylactic vaccine candidates. Conversely, the inherent ability to control TB disease progression makes the CCM model an ideal system for the study of chronic disease states and the evaluation of post-exposure vaccination strategies, and the capacity tolerate high bacterial and disease burden is an attribute that can be utilised for the evaluation drug compounds and therapeutic strategies.

## Supplementary Information

Supplementary Information.

## Data Availability

All data generated or analysed during this study are included in this published article (and its Supplementary Information files).
