# Supplementary material for: High-dose Mycobacterium tuberculosis aerosol challenge cannot overcome BCG-induced protection in Chinese origin cynomolgus macaques; implications of natural resistance for vaccine evaluation
Source: Sci Rep. 2021 Jun 10;11:12274. doi: 10.1038/s41598-021-90913-0 (PMC8192909; doi:10.1038/s41598-021-90913-0)
Supplement: Supplementary file 1 — Supplementary Information. [file 41598_2021_90913_MOESM1_ESM.docx]

**High-dose *Mycobacterium tuberculosis* aerosol challenge cannot overcome BCG-induced protection in Chinese origin cynomolgus macaques; implications of natural resistance for vaccine evaluation**

Laura Sibley^1*^, Andrew D White^1^, Karen E Gooch^1^, Lisa M Stevens^1^, Rachel Tanner^2^, Ashley Jacobs^3^, Owen Daykin-Pont^1^, Fergus Gleeson^4^, Anthony McIntyre^4^, Randall Basaraba^5^, Simon Clark^1^, Graham Hall^1^, Geoff Pearson^1^, Emma Rayner^1^, Helen McShane^2^, Ann Williams^1^, Mike Dennis^1^, Philip D Marsh^1^, Sally Sharpe^1^.

^1^Public Health England, National Infection Service, Porton Down, Wiltshire, SP4 0JG, UK.

^2^The Jenner Institute, Nuffield Department of Medicine, University of Oxford, Oxford, UK

^3^University of Cape Town, Cape Town, South Africa

^4^Churchill Hospital, Headington, Oxford, UK

^5^Colorado State University, Fort Collins, Colorado, USA

*Corresponding author: Tel +441980 619864

Email: laura.sibley@phe.gov.uk

Key words

Tuberculosis, BCG, vaccine, non-human primate, aerosol challenge*, Mycobacterium tuberculosis, monocyte:lymphocyte ratio*

Running title, BCG protects cynomolgus macaques against aerosol *M. tuberculosis* challenge

**Supplementary data**

**
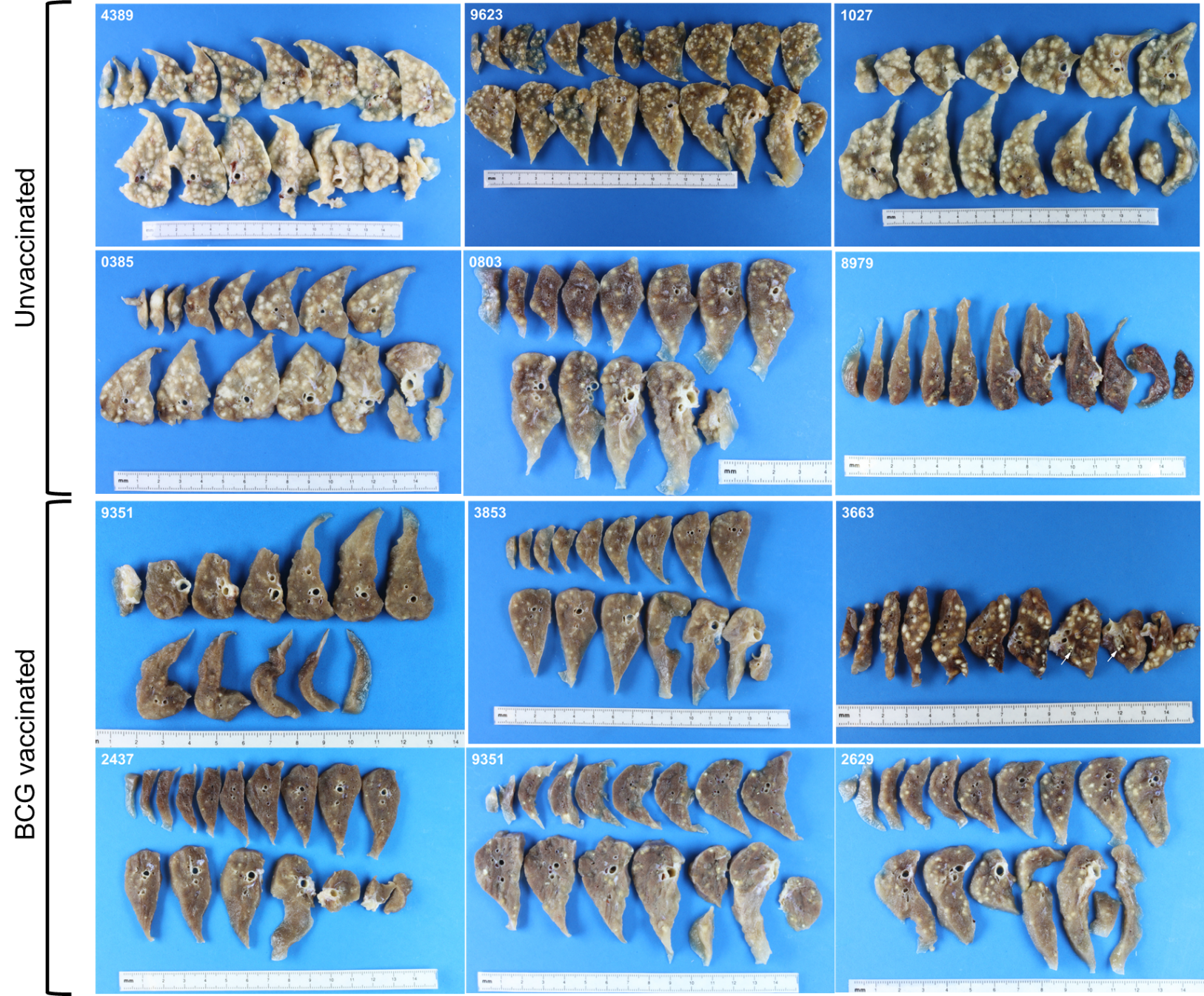
**

**Supplementary data 1. Representative images of lung lobes of unvaccinated and BCG vaccinated macaques after infection with *M. tb*.**

**Supplementary figure 2. *M. tb* cultured from tissue homogenates.** Triangles indicate animals that met humane end point criteria. White = unvaccinated, black = BCG vaccinated.


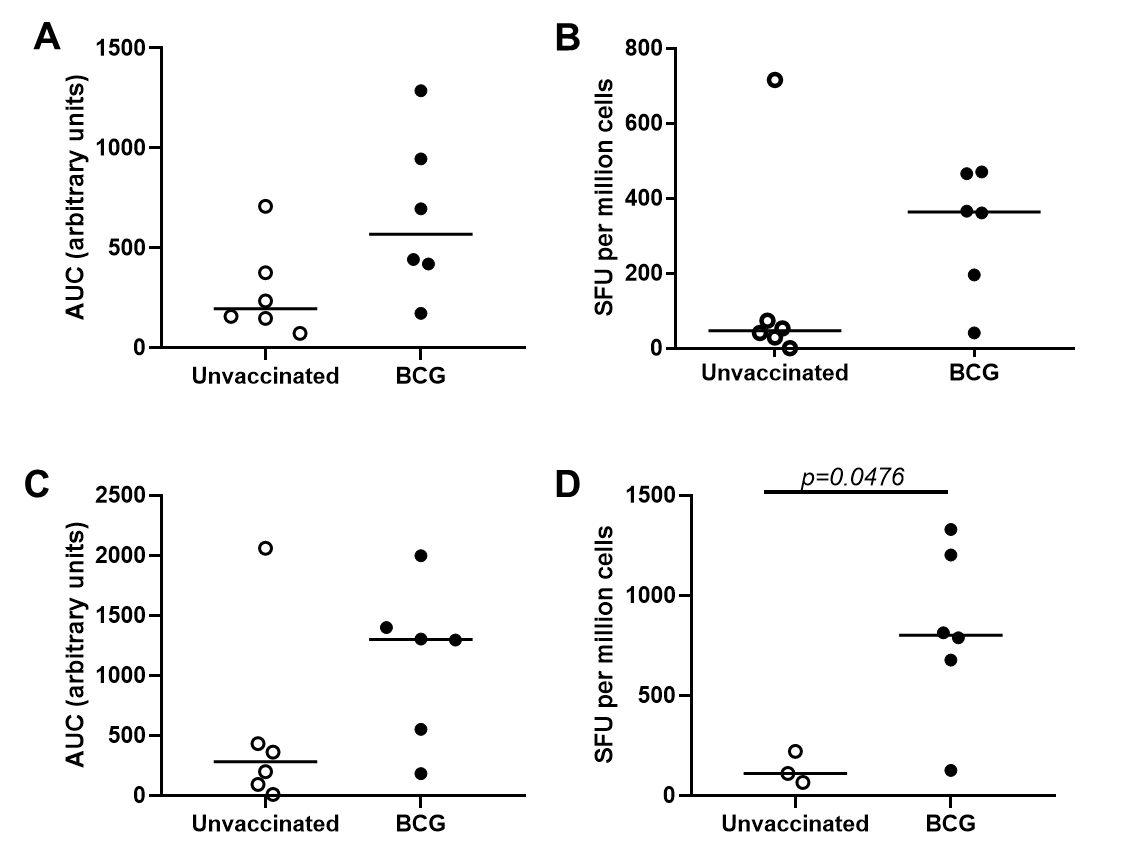


**Supplementary data 3.** PPD-specific ELISPOT analysis. A) AUC of the vaccination phase, B) SFU at week 2 post-infection, C) AUC week 20-25, D) SFU at week 12 post-infection (week 33).


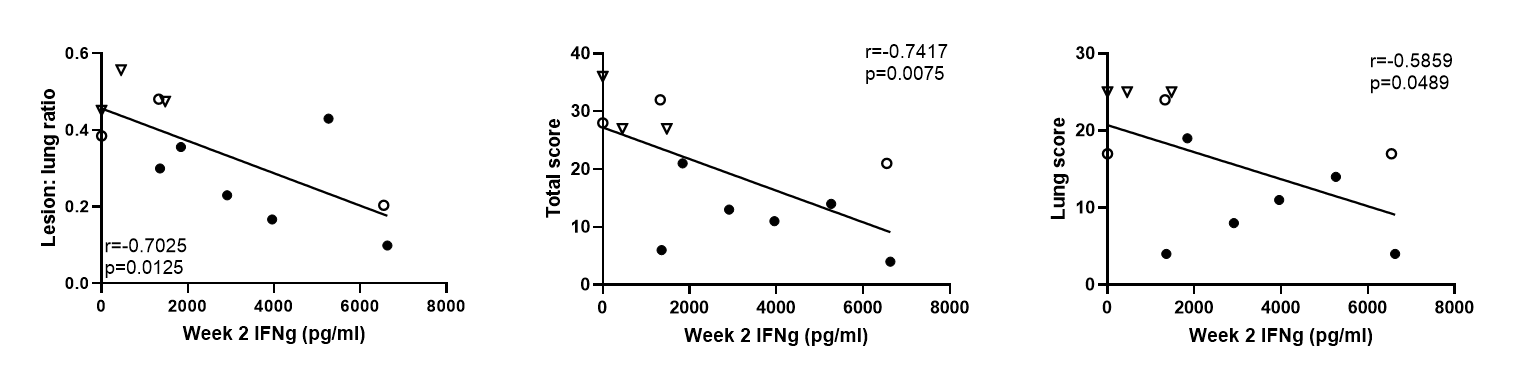


**Supplementary data 4**. Correlations between disease readouts with IFNγ measured in stimulated whole blood supernatants as measured by ELISA


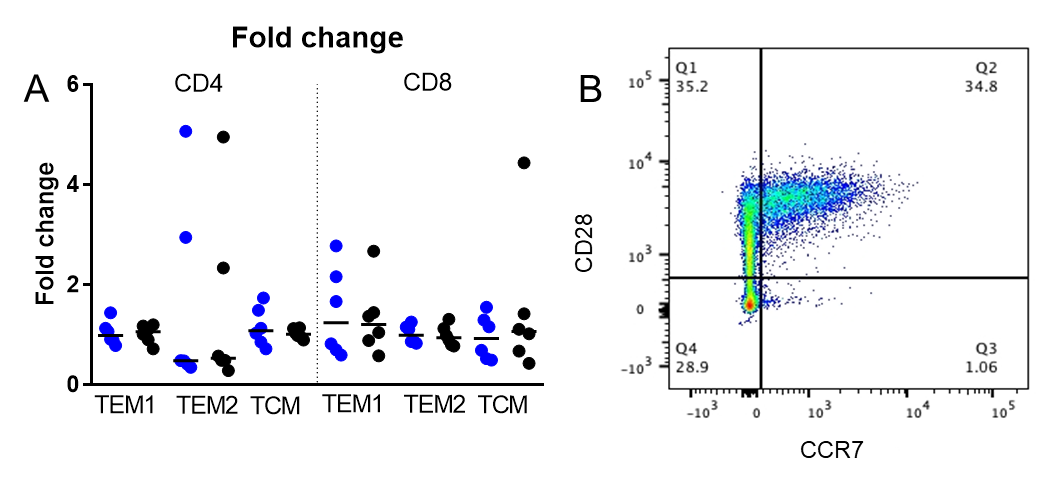


**Supplementary data 5. Analysis of memory populations in CCM at week 20 post-vaccination and comparison to RM memory populations at baseline.** A) Fold change in TEM1, TEM2 and TCM populations between baseline and week 20 post-vaccination in BCG vaccinated (blue) and control animals (black) in CD4+ and CD8+ T-cells. B) Flow cytometry plot showing the staining pattern which determines TEM1 (CCR7^-^ CD28^+^), TCM (CCR7^+^ CD28^+^) and TEM2 (CCR7^-^ CD28^-^) populations in CCM.

**Supplementary data 6: cell population data from the haematology analyser (IDEXX).** Cell counts of lymphocytes, monocytes, neutrophils and eosinophils post vaccination and up to week 12 post-infection. Comparison of lymphocytes, monocytes and granulocytes at week 4 post-infection.

**Supplementary data 7: correlation of M:L at baseline with total pathology score.**

**Supplementary data 8: Monocyte subsets defined using CD14^+^ and CD16^+^ in PBMCs at baseline.** Classical monocytes (CD14^+^ CD16^+^), intermediate (CD14^+^ CD16^+^) and non-classical (CD14^-^ CD16^+^).
